# Follicle-Targeted Delivery of Betamethasone and Minoxidil Co-Entrapped in Polymeric and Lipid Nanoparticles for Topical Alopecia Areata Treatment

**DOI:** 10.3390/ph16091322

**Published:** 2023-09-19

**Authors:** Breno N. Matos, Ana Luiza Lima, Camila O. Cardoso, Marcilio Cunha-Filho, Tais Gratieri, Guilherme M. Gelfuso

**Affiliations:** 1 Laboratory of Food, Drugs, and Cosmetics (LTMAC), University of Brasília, Brasilia 70910-900, DF, Brazil; brenomatos15@hotmail.com (B.N.M.); anaaluiza.ln@gmail.com (A.L.L.); ca_mila.oc@hotmail.com (C.O.C.); marciliofarm@hotmail.com (M.C.-F.); tgratieri@gmail.com (T.G.); 2 School of Heath Sciences, Campus Universitário Darcy Ribeiro, s/n, Brasilia 70910-900, DF, Brazil

**Keywords:** hair follicles, drug delivery, nanotechnology, skin delivery

## Abstract

Alopecia areata is managed with oral corticosteroids, which has known side effects for patients. Given that a topical application of formulations containing a corticoid and a substance controlling hair loss progression could reduce or eliminate such adverse effects and increase the patient’s adherence to the treatment, this study prepares polymeric and lipidic nanoparticles (PNPs and NLCs) to co-entrap minoxidil and betamethasone and compares the follicular drug delivery provided by topical application of these nanoparticles. The prepared PNPs loaded 99.1 ± 13.0% minoxidil and 70.2 ± 12.8% betamethasone, while the NLCs entrapped 99.4 ± 0.1 minoxidil and 80.7 ± 0.1% betamethasone. PNPs and NLCs presented diameters in the same range, varying from 414 ± 10 nm to 567 ± 30 nm. The thermal analysis revealed that the production conditions favor the solubilization of the drugs in the nanoparticles, preserving their stability. In in vitro permeation studies with porcine skin, PNPs provided a 2.6-fold increase in minoxidil penetration into the follicular casts compared to the control and no remarkable difference in terms of betamethasone; in contrast, NLCs provided a significant (specifically, a tenfold) increase in minoxidil penetration into the hair follicles compared to the control, and they delivered higher concentrations of betamethasone in hair follicles than both PNPs and the control. Neither PNPs nor NLCs promoted transdermal permeation of the drugs to the receptor solution, which should favor a topical therapy. Furthermore, both nanoparticles targeted approximately 50% of minoxidil delivery to the follicular casts and NLCs targeted 74% of betamethasone delivery to the hair follicles. In conclusion, PNPs and NLCs are promising drug delivery systems for enhancing follicular targeting of drugs, but NLCs showed superior performance for lipophilic drugs.

## 1. Introduction

Alopecia areata is an immune-mediated disease characterized by hair loss that can occur at various parts of the body, including the scalp, eyebrows, and eyelashes [1]. The disease affects approximately 2% of the global population [2] and is primarily found among individuals aged 25–36 years [3,4]. Current treatments for alopecia areata are not curative but rather aim to halt the disease’s progression. The main strategy involves using high doses of corticosteroids to control the autoimmune causes, and topically applied minoxidil may also be prescribed to prevent hair loss progression [5,6]. However, there is currently no formulation, either oral or topical, available on the market that combines both substances into a single formulation, which would potentially enhance treatment adherence.

Although the off-label use of minoxidil in oral doses of 1 mg has been proposed for alopecia treatment, regulatory agencies (including the FDA) only approve the topical use of this drug, due to its hypotensive effects [7]. In addition, systemic use of corticosteroids is associated with a range of undesirable side effects, such as weight gain, fluid retention, edema, elevated blood pressure, slow wound healing, osteoporosis, and adrenal gland suppression, the latter of which can result in adrenal insufficiency upon discontinuation of the medication [8]. Therefore, topical administration of the two substances in a single formulation to treat alopecia areata remains a safer option for patients [9]. Nevertheless, the effectiveness of this therapy is directly related to the penetration of drugs in effective quantities reaching the therapeutic target—the hair bulbs.

This work proposes the co-encapsulation of minoxidil and the corticoid betamethasone in nanoparticles for topical application. The rationale for this approach is that, in addition to guaranteeing formulation stability by incorporating two drugs of different polarities, these nanoparticles could target drug delivery to hair follicles. Numerous studies have demonstrated that topically applied nanoparticles can naturally accumulate in the hair follicles [10,11,12,13,14,15,16], providing targeted drug delivery to this site and increasing the treatment efficacy. Moreover, any proposed formulations should avoid adding organic solvents that could cause specific toxic effects [10].

Although there is consensus on the follicular accumulation of topically applied nanoparticles, the effect of system characteristics (such as composition) in promoting more effective follicular penetration and thus ensuring a greater targeting of the encapsulated drugs’ delivery into the follicles is still not well-established. Therefore, this work evaluated polymeric nanoparticles (PNPs) and nanostructured lipid carriers (NLCs). Polycaprolactone was selected for the preparation of PNPs, as it is a semi-crystalline, hydrophobic, biocompatible, biodegradable polymer with a low degradation rate [12,17]. In addition, other works have already shown that this polymer can load high rates of minoxidil [18,19] and betamethasone [17,20].

NLCs are lipid-based nanoparticles that offer numerous advantages in the field of dermatology. They are composed of a solid lipid matrix and a liquid lipid: the solid lipid provides stability and rigidity to the carrier, while the liquid lipid improves drug loading capacity and enhances drug-release kinetics [21]. Due to their lipid composition, NLCs are biocompatible with and possibly have an affinity with the sebaceous content secreted inside hair follicles, which may favor their accumulation in these dermal structures, as previously inferred [14,16].

In short, this study prepares polymeric and lipidic nanoparticles to co-entrap minoxidil and betamethasone, and it compares the follicular drug delivery provided by the different systems, aiming to establish an efficient topical treatment for alopecia areata.

## 2. Results and Discussion

### 2.1. Development and Characterization of the Nanoparticles

PNPs and NLCs containing minoxidil and betamethasone were successfully prepared and presented as low-opacity colloidal dispersions. After obtaining them, the systems were subjected to characterization tests to determine the mean size and size distribution of the formed nanoparticles, the zeta potential, and the entrapment efficiency of each co-encapsulated drug in the systems. The results are presented in Table 1.

Both nanoparticles without drugs showed similar mean hydrodynamic diameters (270.7 ± 16.8 nm for PNPs and 272.0 ± 3.2 nm for NLCs). However, in both cases, the incorporation of the drugs considerably increased the nanoparticles’ diameter, with the change being more significant for NLCs (1.5-fold for PNPs and 2-fold for NLCs). This increase in size was expected, since it indicates the drug loading, as observed in previous studies with polymeric [22,23] and lipid [24] nanoparticles. The polydispersity index (PdI), which was less than 0.23 for all nanoparticles, indicated that monodispersed systems were developed independently of the matrix used for entrapping the drugs [23].

All nanoparticles exhibited zeta potential values below −20 mV once the polymer and the lipid material possessed carboxyl groups [25,26]; however, the nanoparticles containing the drugs displayed a more negative charge (−32.1 ± 2.9 and −37.9 ± 0.02 mV versus −20.7 ± 0.5 and −21.26 ± 0.4). This slight increase in negative charge may be attributed to phosphate counter-ions from betamethasone [24,27], which must be adsorbed on the nanoparticles’ surface [28]. These zeta potential values can indicate greater physical stability of the dispersed nanoparticles, due to electrostatic repulsion [24,29].

The entrapment efficiency was determined by assessing the extent to which the drug interacted with the nanoparticles, either inside or attached to their surface. The efficiency values for PNPs were 99.1 ± 13.0% for minoxidil and 70.2 ± 12.8% for betamethasone; for NLCs, the efficiency values were 99.4 ± 0.1% for minoxidil and 80.7 ± 0.1% for betamethasone. These high values were expected since both polymers and lipids as well as the drugs are lipophilic. In fact, the log P of the drugs (1.2 for minoxidil and 3.6 for betamethasone phosphate [30]) not only indicates the lipophilicity of the drugs but also explains the encapsulation being higher than 99.1% for minoxidil and 70.2% for betamethasone, regardless of the nanoparticles’ composition.

It is worth mentioning that since the primary purpose of the present work is to compare the performance of the two nanoparticles in follicular drug targeting with topical application, the fact that both nanoparticles have similar sizes (ranging from 400 to 600 nm) and drug entrapment efficiencies that are statistically identical for minoxidil (*p* < 0.0001) and betamethasone (*p* < 0.0001) enables the interpretation of the drugs’ skin permeation results.

**Figure 1:**
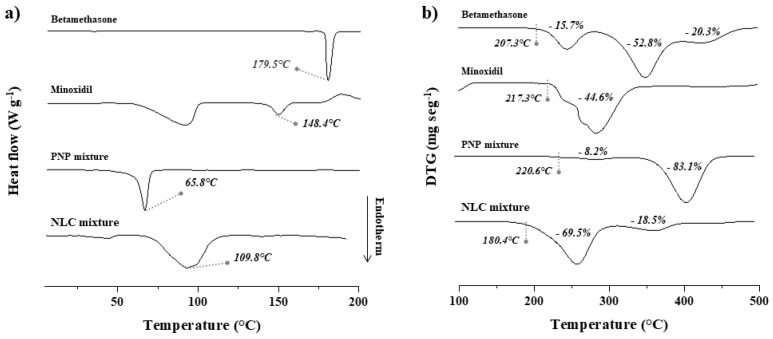
(**a**) DSC and (**b**) TGA curves of betamethasone and minoxidil as supplied and in PNP and NLC mixtures, which are the drug excipients’ physical mixture of each nanoformulation. For DSC analysis, the peak temperature is indicated; for TGA analysis, the initial degradation temperature is indicated, and the respective mass loss of each event is shown in percentage (%).

DSC curves are presented in Figure 1a: betamethasone showed an endothermic peak at 179 °C and minoxidil showed one at 143.5 °C, corresponding to their respective melting temperatures. In the sample containing polycaprolactone (PNP mixture), the melting peak of both drugs completely disappears. Thus, after the polymer is melted (an endothermic event that occurs at 65.8 °C; Figure 1a), both minoxidil and betamethasone seem solubilized in the melted polymer.

For the NLC mixture, a wide endotherm involving high enthalpy was observed from 75 to 110 °C (Figure 1a). This thermal event has no direct correspondence with the mixture’s components; it is possible that this peak is related to the melting of stearic acid added to a strong thermal interaction with drugs that do not present their characteristic melting events. With the potential of sample decomposition in this temperature range removed (as confirmed by the TGA results), it can be inferred that crystalline drugs are largely solubilized in NLC components [31].

Thermogravimetric analyses (TGA) are presented in Figure 1b by their first derivative, in which the thermal degradations of betamethasone and minoxidil were identified to begin at 207.3 °C and 217.3 °C, respectively. In the case of the PNP mixture, the initial degradation temperature was practically unchanged, and most of the mixture degraded from 341 °C (Figure 1b). For the NLC mixture, the anticipation of thermal degradation was observed, which was expected, since the TGA of the lipid excipients as supplied showed that these materials degrade near 180 °C. These findings indicate that the materials combined in both formulations are thermally compatible. Moreover, the initial degradation temperature observed in derivative thermogravimetric (DTG) curves is significantly lower than the thermal region explored in the production protocol for PNPs and NLCs, which involves heating the materials at 40 and 80 °C, respectively. Thus, the thermal analysis led to the conclusion that both minoxidil and betamethasone present solubility in the polymeric and lipid materials that is favorable at moderate temperatures. Such a temperature range used in formulation production has been shown to be safe for nanoparticles’ development [32,33].

### 2.2. In Vitro Drug Release

The release of each drug from the studied nanosystems was compared to the free drugs’ diffusion through cellulose membrane (control), which was used as a support for the in vitro experiments.

**Figure 2:**
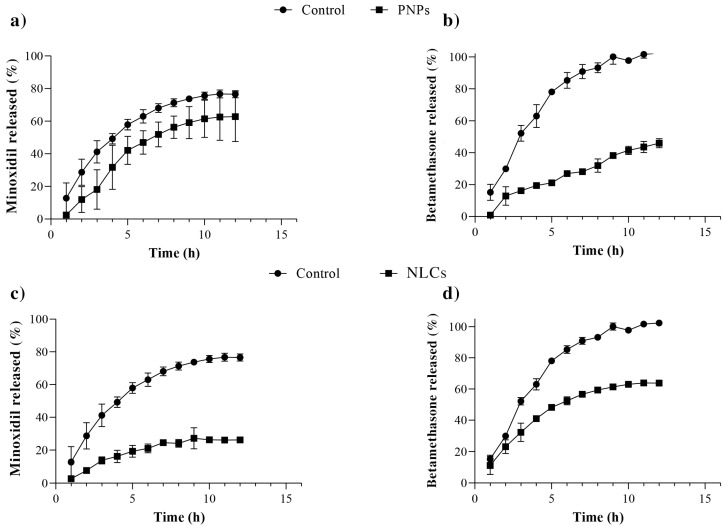
Comparative release profiles of minoxidil (**a**,**c**) and betamethasone (**b**,**d**) from drug loaded-PNPs (**a**,**b**) and NLCs (**c**,**d**) or aqueous solution of the drugs (control). The experiments were conducted six times for each formulation (n = 6). All drug release profiles fitted the Higuchi kinetics model (r > 0.9) except betamethasone release from PNPs, which fitted a zero-order model.

As can be seen in the results shown in Figure 2, PNPs do not appear to control minoxidil release (*p* > 0.05) effectively: in the control group, approximately 80 ± 2% of the minoxidil content crossed the membrane into the PBS solution after 12 h of the experiment, while 63 ± 15% of the drug was released from the PNPs during the same period (Figure 2a). In contrast, NLCs exhibited a reduced minoxidil release, with only 27 ± 1% of the drug being released over 12 h (Figure 2c). The lipophilic nature of the drug may keep it dissolved within the inner matrix, which is composed of hydrophobic long-chain fatty acids [14,16,34,35,36,37]. Overall, these results show that NLCs have a more pronounced ability than PNPs to retain minoxidil within the formulation.

With respect to betamethasone, PNPs demonstrated controlled drug release, with only 46 ± 3% of the drug quantified in the receptor solution after 12 h (Figure 2b). NLCs demonstrated a significantly higher release profile, with 64 ± 3% of the drug being released during the same period (Figure 2d). It is worth noting that betamethasone, despite being slightly less fat-soluble than minoxidil (log *p* values of 1.24 for minoxidil and 1.13 for betamethasone [30]), exhibited a faster release rate, probably due to different interactions with the NLCs’ composition lipids [34].

Despite the different release profiles observed for these drugs, the lipid nanocarriers reduced the releases of minoxidil and betamethasone by approximately 2.8-fold and 1.6-fold, respectively, compared to the control group (*p* ≤ 0.0001 for both comparisons), throughout the 12 h experiment.

### 2.3. Skin Permeation Studies

**Figure 3:**
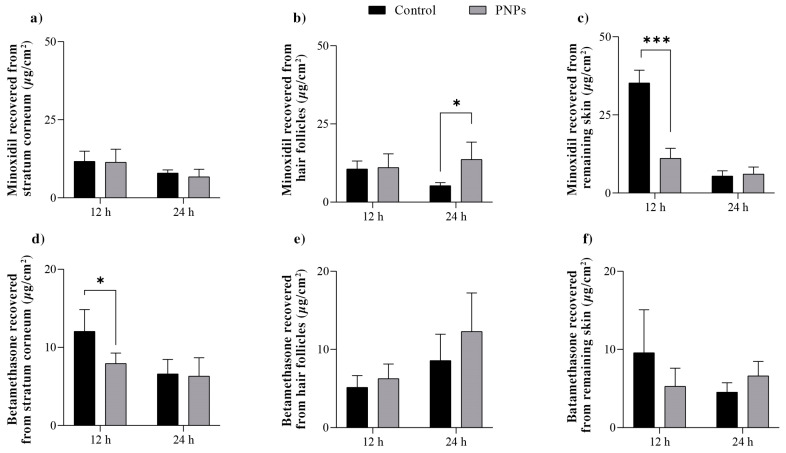
Minoxidil (**a**–**c**) and betamethasone (**d**–**f**) recovered from the stratum corneum (**a**,**d**), hair follicles (**b**,**e**), and remaining skin (**c**,**f**) after 12 and 24 h of topical treatment with the PNPs or the aqueous solution of the drugs (control). The experiments were conducted six times for each formulation and time-point (n = 6). Statistical significance is indicated by * *p* < 0.05 and *** *p* < 0.005.

The skin penetration of minoxidil and betamethasone from PNPs compared to that from the free-drug solution was assessed for 12 and 24 h. The results are presented in Figure 3.

In none of the skin permeation experiments were detectable concentrations of both minoxidil and betamethasone verified, regardless of the analyzed formulation. Given that the analytical validity method is sensitive, this indicates that the formulations could concentrate the drug penetration in the skin layers, avoiding systemic exposure, which is desirable for a topical formulation.

The results demonstrate that PNPs did not affect minoxidil penetration into the stratum corneum compared to the control, regardless of the analyzed time point. However, a significant difference was observed in the drug penetration into deeper layers of the skin. After 24 h of treatment, the PNPs yielded a 2.6-fold increase (*p* < 0.05) in minoxidil penetration in the follicular casts over the control, along with a 3.2-fold decrease (*p* < 0.005) in the concentration of the drug deposited in the remaining skin after the first 12 h of experiment.

In terms of the betamethasone penetration, at 12 h of the experiment, the nanoparticles significantly reduced the amounts penetrating the stratum corneum (*p* < 0.05), which may be a result of the release control exerted by the nanoparticles, and did not show any significant alteration in the drug’s penetration into the hair follicles or the remaining skin compared to the control. This situation was maintained after 24 h.

**Figure 4:**
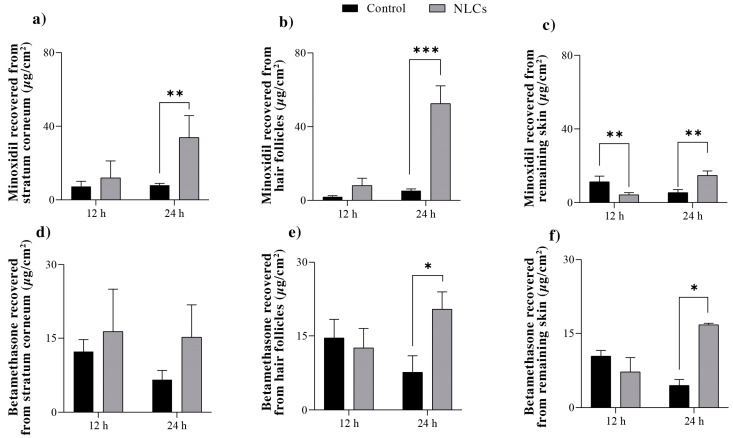
Minoxidil (**a**–**c**) and betamethasone (**d**–**f**) recovered from the stratum corneum (**a**,**d**), hair follicles (**b**,**e**), and remaining skin (**c**,**f**) after 12 and 24 h of topical treatment with the NLCs or the aqueous solution of the drugs (control). The experiments were conducted six times for each formulation and time-point (n = 6). Statistical significance is indicated by * *p* < 0.05, ** *p* < 0.005, *** *p* < 0.005.

The skin penetration of minoxidil and betamethasone from the NLCs compared to that from the free-drug solution was also assessed at 12 and 24 h, with the results presented in Figure 4.

The amount of minoxidil quantified in the stratum corneum after 12 and 24 h was comparable when using NLCs or the control, with an observed accumulation of the drug in this skin layer over time. After 24 h of treatment, a 10-fold increase (*p* < 0.005) in follicular penetration of minoxidil was observed with the application of NLCs compared to that of the control. This greater drug penetration with NLCs was also noted in the remaining skin after 24 h, but in a smaller magnitude—only 2.7-fold compared to the control.

With regard to betamethasone, NLCs did not provide any difference in the penetration in the stratum corneum or the remaining skin compared to the control, regardless of the exposure time. However, NLCs more than doubled (*p* < 0.005) the concentration of betamethasone accumulated in the hair follicles after both 12 and 24 h of topical treatment.

Thus, in a comparison of the effect of the two types of nanoparticles on the follicular penetration of the drugs, although both increased the penetration after 24 h of topical treatment, the NLCs were notably more efficacious in increasing the drugs’ accumulation in the follicular structures.

If we consider other similar published studies, a polycaprolactone nanocapsule of latanoprost (a lipophilic drug) did not significantly increase follicular penetration of the drug compared to the control and only provided a 2-fold increase in drug follicular penetration after the nanoparticles were massaged into the skin [37]. In contrast, NLCs containing the same drug provided a significant 3-fold increase in drug follicular penetration without massage application [16].

The effect of the nanoparticles in the current study on follicle-targeting drug delivery can be better comprehended through an analysis of the follicle targeting factor. This factor considers the percentage of drugs penetrating the hair follicles compared to the total amount of drugs penetrating the skin from a given formulation.

Data from Table 2 show that after 24 h of skin exposure to the three tested formulations (control/free-drug solution, PNPs, and NLCs), both nanoparticles targeted approximately 50% of minoxidil delivery to the follicular casts (2-fold higher than the control). Regarding betamethasone, the PNPs targeted a similar proportion of the drug to the hair follicles as the control did (43% and 49%, respectively), while the NLCs targeted approximately 74% of betamethasone delivery to the hair follicles.

This result confirms the potential of nanoparticles to naturally direct the release of drugs to follicular structures; in the case of a disease that affects these structures—such as androgenic alopecia—this dramatically benefits the topical treatment. The study also reveals the more significant potential of NLCs to promote such targeting, most likely due to the greater affinity of the lipids comprising these nanoparticles with the sebaceous content secreted within the hair follicles [14,16,24,38,39,40].

On a final note, it is important to highlight that the analytical method used to quantify both drugs extracted from the skin was validated in accordance with the ICH guidelines [41]. The method proved to be selective and linear, with linear correlation coefficients of 0.9996 and 0.9924 for minoxidil and betamethasone, respectively. The limits of detection and quantification for minoxidil were 0.21 and 0.64 µg/mL, respectively, and those for betamethasone were 0.04 and 0.13 µg/mL, respectively. The recovery efficiencies of minoxidil and betamethasone from the stratum corneum, follicular casts, and remaining skin were high, with values of 98.2 ± 0.1%, 97.7 ± 0.1%, and 97.9 ± 0.1% for minoxidil and 96.4 ± 0.1%, 106.5 ± 0.1%, and 97.2 ± 0.1% for betamethasone, respectively.

## 3. Material and Methods

### 3.1. Material

The minoxidil (3-hydroxy-2-imino-6-piperidin-1-ylpyrimidin-4-amine, >99%) used in this work was acquired from SM Empreendimentos Farmacêuticos (Anápolis, Brazil). Betamethasone phosphate {2-[(1R,2S,3aS,3bS,9aS,9bR,10S,11aS)-9b-fluoro-1,10-dihydroxy-2,9a,11a-trimethyl-7-oxo-1H,2H,3H,3aH,3bH,4H,5H,7H,9aH,9bH,10H,11H,11aH-cyclopenta[a]phenanthren-1-yl]-2-oxoethoxy}phosphonic acid > 99%) was acquired from Aché (São Paulo, Brazil). Acetone, polycaprolactone (MW = 2000 g/mol, 2-[2-(6-hydroxyhexanoyloxy)-ethoxy]-ethyl-6-hydroxyhexanoate), Tween80^®^, oleic acid, and stearic acid were acquired from Sigma-Aldrich (Steinheim, Germany). Soy lecithin (Lipoid^®^S 100) was purchased from Lipoid (Ludwigshafen, Germany). Acetonitrile and methanol of HPLC grade were acquired from J.T. Barker (Philipsburg, PA, USA). All analyses were performed with ultrapure water (Millipore, Illkirch-Graffenstaden, France). Scotch storage packing long-lasting and cyanoacrylate glue from 3M (St Paul, MN, USA) was used to process the skin after the cutaneous permeation studies, and cellulose acetate membranes (MW 12,000–14,000) purchased from Fisher (Pittsburg, KS, USA) were used in the release studies.

### 3.2. Skin

Porcine ear skin was used in in vitro permeation experiments. The ears were obtained from a local slaughterhouse (Frigorifico Via Carnes) less than 2 h postmortem of the animal. The whole skin was removed from the outer region of the ear and separated from its underlying layer, and “full thickness” was used to guarantee the intactness of the hair follicles. The skin was frozen at −20 °C for a maximum of 1 month before use.

### 3.3. Development of the PNPs

The PNPs were obtained using a method adapted from the literature [42]. Briefly, 0.075 g polycaprolactone was dissolved in 7.5 mL acetone under magnetic stirring (300 rpm) at 30 °C until complete solubilization. A mass ratio corresponding to 2% *w*/*v* minoxidil and 0.1% *w*/*v* betamethasone was then dissolved in this same phase, based on the final volume of the formulation. Simultaneously, the aqueous phase was prepared by dissolving 50 mg Tween80^®^ in 10 mL water. The organic phase was then gradually poured into the aqueous phase under moderate magnetic stirring via a syringe. The dispersion was subsequently transferred to a rotary evaporator (IKA RV 10, Brazil) for extraction of all the organic solvent (solvent extraction was confirmed by analysis of the final volume of the formulation). Afterward, the resulting nanoparticles were collected and stored in a refrigerator at 4 °C until further use.

A similar procedure was followed for preparing PNPs without the drug to be used as a control, except the drugs were not added to the initial organic phase containing polycaprolactone.

### 3.4. Development of the NLCs

The NLCs were prepared using the microemulsion dispersion technique. A mixture consisting of an oily phase (oleic acid and stearic acid) and the surfactants (Tween 80^®^ and soy lecithin) was heated to 80 °C with moderate stirring (300 rpm) to ensure complete fusion of the components. The mass ratio—corresponding to 2% *w*/*v* minoxidil and 0.1% *w*/*v* betamethasone based on the final volume of the formulation—was then solubilized at the lipid composition. Subsequently, 500 µL of water heated to 80 °C was added, and the system was maintained in a state of constant agitation (500 rpm) for 10 min to form a microemulsion. Thereafter, the microemulsion was dispersed into an aqueous phase under vigorous stirring using an Ultra-Turrax (IKA T18, Brazil) at 13,000 rpm for 5 min while being kept in an ice bath (± 5 °C). To reduce the nanoparticles’ size, the dispersion was subjected to sonication, operated at 20% amplitude with continuous pulses for 5 min.

A similar procedure was followed for preparing NLCs without the drug to be used as a control, except the drugs were not added to the lipid phase.

### 3.5. Characterization of the Nanoparticles

#### 3.5.1. Hydrodynamic Diameter and Zeta Potential

The hydrodynamic diameter distribution of the nanoparticles and the zeta potential were analyzed through dynamic light scattering and electrophoretic mobility techniques, respectively. To this end, a 1 mL sample of each nanoparticle’s suspension (with or without the drugs) was analyzed in a Zetasizer Nano ZS (Malvern, Worcestershire, UK).

#### 3.5.2. Entrapment Efficiency

The entrapment efficiency was determined by centrifuging the nanoparticles’ suspension in a device with a pore size set at 10 kDa (Vivaspin 2, 100,000, MWCO HY, Sartorio, Goettingen, Germany). The samples were centrifuged for 10 min at 4000 rpm in a centrifuge (KASVI, Brazil). The difference between the initially added drug concentration (FT) and the obtained unentrapped drug concentration (FL) determined the entrapped drug concentration. Thus, the entrapment efficiency (EE%) was calculated as follows:

EE% = [(FT − FL)/FT] × 100(1)

#### 3.5.3. Thermal Analyses

Thermal analysis techniques—specifically, differential scanning calorimetry (DSC) and thermogravimetric analysis (TGA)—were used to characterize the samples. Physical mixtures of the formulation components were prepared in equal mass-to-mass proportion in order to maximize the compounds’ events and enable a better interpretation of the results [43,44].

Each mixture containing the components used in PNPs and NLCs was carefully mixed with a mortar and pestle. For the PNP formulation, betamethasone, minoxidil, and polycaprolactone were combined; for the NLC formulation, betamethasone, minoxidil, stearic acid, oleic acid, and soy lecithin were combined. Additionally, analyses were also performed of the drugs and excipients as supplied, for better comparison.

DSC analyses were performed with a DSC-60 (Shimadzu, Kyoto, Japan) operating at a heating rate of 10 °C min^−1^ from 25 to 200 °C with 3–5 mg samples placed in aluminum pans. TGA was performed with a DTG-60H (Shimadzu, Kyoto, Japan) operating at a heating rate of 10 °C min^−1^ from 25 to 500 °C with 3–5 mg samples placed in platinum pans. All analyses were performed in a nitrogen atmosphere at a flow of 50 mL min^−1^. DSC and TGA analyses were conducted in a nitrogen atmosphere at a 50 mL min^−1^ flow. All thermal measurements were calculated using the TA-60 software (Shimadzu, Kyoto, Japan), and the curves obtained by TGA were represented by its first derivative.

### 3.6. In Vitro Drug Release

Drug release from the nanoparticles was assessed using Franz diffusion cells set up with cellulose acetate membranes between donor and receptor compartments. The receptor compartment of the cells was filled with 15 mL of a pH 7.4 phosphate buffer solution containing 0.5% Tween80^®^ and was maintained at 37 °C using Hanson’s DB-6 dry heat model equipment. In the donor compartment, 1 mL of each nanoparticle’s suspension (PNP or NLC) was added. Throughout the 12 h experiment, 1 mL of the receiving solution was collected for high-performance liquid chromatography (HPLC) analysis every 1 h, at which time the solution in the receptor compartment was replenished.

To rule out the effect of the membrane on drug release, a similar experiment was conducted by placing 1 mL of an aqueous solution containing the same concentration of the drugs in the donor compartment as a control. Each formulation was tested six times (n = 6). The drug release profile of the nanoparticles was analyzed and compared with that of the aqueous drug solution, using graphical methods that plotted the percentage of drug released over time.

### 3.7. Skin Permeation Studies

These studies were conducted in vitro using Franz diffusion cells set up with porcine ear skin between the donor and receptor compartments. The receptor compartment was filled with a pH 7.4 phosphate buffer solution containing 0.5% Tween80^®^. The temperature was maintained at 37 °C throughout the experiment using Hanson’s DB-6 dry heat model equipment. The donor compartment was filled with 1 mL of the nanoparticle’s formulations or of the control (prepared as described in 3.6). Each formulation was tested six times (n = 6).

The receptor solution was continuously stirred at 300 rpm, and independent studies were conducted at 12 h and 24 h for each formulation.

After each experiment, samples of the receptor media were collected and analyzed using a validated HPLC method. The skin was then removed from the diffusion cells, gently cleaned with a water-soaked gauze pad, dried, and placed on a flat surface with the stratum corneum facing up. At this point, the skin was subjected to 15 tape-stripping cycles. The tapes were placed in a flask containing 5 mL methanol and left under moderate stirring over a 24 h period to recover the minoxidil and betamethasone content. A drop of cyanoacrylate superglue was applied to the stripped skin area and then covered with another tape strip (applied using light pressure) to obtain additional information about drug distribution within the skin. After complete polymerization of the glue (approximately 5 min), this tape was removed, and the skin surface biopsy obtained herein contained follicular casts. Minoxidil and betamethasone were extracted from these casts using 5 mL methanol and quantified. Finally, the remaining skin was chopped to maximize its surface area and was subjected to exhaustive extraction in methanol for 24 h, followed by drug quantification.

Data are presented as the amount of drug recovered from each skin layer divided by the area of formulation application (µg/cm^2^) for each formulation and experimental time. In addition, a “follicle targeting factor” for each formulation was calculated by dividing the concentration of the drug recovered from the hair follicles (HF) by the combined concentration of the drug recovered from the entire skin (stratum corneum, SC; hair follicles and remaining skin, RS) [16]:

Targeting Factor = HF/(SC + HF + RS)(2)

### 3.8. Minoxidil and Betamethasone Quantification

Minoxidil and betamethasone phosphate were quantified using the HPLC system Shimadzu LC 20-AD model, consisting of two pumps (LC 20-AT model), an automatic injector (9SIL-20AD model), an oven (CTO-20AS model), a spectrophotometric detector (SPD-M20A model), and a computer equipped with the Shimadzu LC chromatographic analysis program. A reverse-phase C_18_ column (150 mm × 4.6 mm) was used as the stationary phase. The mobile phase was composed of a mixture of ultrapure water and acetonitrile (53:47 *v*/*v*), which eluted in a gradient that rose to 45:55 *v*/*v* at t = 8 min and returned to 53:47 *v*/*v* at t = 12 min. The flow rate was 1 mL/min, and the sample injection volume was 20 μL. The oven was used at a temperature of 40 °C, and detection was performed at 285 nm for minoxidil and 245 nm for betamethasone.

### 3.9. Data Analysis

The results are presented as means ± standard deviation of at least three replicates. The data were analyzed through two-way ANOVA. The threshold of statistical significance was fixed at *p* < 0.05.

## 4. Conclusions

PNPs and NLCs exhibited considerable potential as topical drug delivery systems, as they effectively enhanced the targeting of minoxidil and betamethasone to hair follicles, without promoting transdermal permeation for any of the drugs in detectable concentrations. Remarkably, NLCs display enhanced efficiency, particularly for more lipophilic drugs. Therefore, this study’s outcomes strongly suggest that these innovative systems hold promise for improving alopecia areata treatment.

## Figures and Tables

**Table 1:** Average hydrodynamic diameter, PdI, and zeta potential of the blank nanoparticles (Blank-PNPs and Blank-NLCs) and those loading 2% (*w*/*v*) minoxidil and 0.1% (*w*/*v*) betamethasone (PNPs and NLCs).

Nanoparticles	Hydrodynamic Diameter (nm)	PdI	Zeta Potential (mV)
Blank-PNPs	270.7 ± 16.8	0.04 ± 0.04	−20.7 ± 0.5
PNPs	413.9 ± 9.6	0.18 ± 0.01	−32.1 ± 2.9
Blank-NLCs	272.0 ± 3.2	0.23 ± 0.04	−21.3 ± 0.4
NLCs	566.7 ± 30.1	0.23 ± 0.20	−37.9 ± 0.1

**Table 2:** Follicle targeting factor calculated for minoxidil and betamethasone recovered from the skin treated for 12 and 24 h, with the nanoformulations compared to the control solutions.

Drug	Time (h)	Formulations		
		Control	PNPs	NLCs
Minoxidil	12	0.18	0.32	0.44
	24	0.28	0.51	0.52
Betamethasone	12	0.19	0.32	0.37
	24	0.43	0.49	0.74

## Data Availability

The data presented in this study are available on request from the corresponding author.
